# Pesticide toxicity: a mechanistic approach 

**DOI:** 10.17179/excli2018-1710

**Published:** 2018-11-08

**Authors:** Volodymyr I. Lushchak, Tetiana M. Matviishyn, Viktor V. Husak, Janet M. Storey, Kenneth B. Storey

**Affiliations:** 1 Department of Biochemistry and Biotechnology, Vasyl Stefanyk Precarpathian National University, 57 Shevchenko Str., Ivano-Frankivsk, 76018, Ukraine; 2 Institute of Biochemistry, Carleton University, 1125 Colonel By Drive, Ottawa, Ontario K1S 5B6, Canada

**Keywords:** bioaccumulation, biotransformation, pollutants, mechanisms, oxidative stress, xenobiotics

## Abstract

Pesticides are known for their high persistence and pervasiveness in the environment, and along with products of their biotransformation, they may remain in and interact with the environment and living organisms in multiple ways, according to their nature and chemical structure, dose and targets. In this review, the classifications of pesticides based on their nature, use, physical state, pathophysiological effects, and sources are discussed. The effects of these xenobiotics on the environment, their biotransformation in terms of bioaccumulation are highlighted with special focus on the molecular mechanisms deciphered to date. Basing on targeted organisms, most pesticides are classified as herbicides, fungicides, and insecticides. Herbicides are known as growth regulators, seedling growth inhibitors, photosynthesis inhibitors, inhibitors of amino acid and lipid biosynthesis, cell membrane disrupters, and pigment biosynthesis inhibitors, whereas fungicides include inhibitors of ergosterol biosynthesis, protein biosynthesis, and mitochondrial respiration. Insecticides mainly affect nerves and muscle, growth and development, and energy production. Studying the impact of pesticides and other related chemicals is of great interest to animal and human health risk assessment processes since potentially everyone can be exposed to these compounds which may cause many diseases, including metabolic syndrome, malnutrition, atherosclerosis, inflammation, pathogen invasion, nerve injury, and susceptibility to infectious diseases. Future studies should be directed to investigate influence of long term effects of low pesticide doses and to minimize or eliminate influence of pesticides on non-target living organisms, produce more specific pesticides and using modern technologies to decrease contamination of food and other goods by pesticides.

## Abbreviations

ALT, alanine aminotransferase; AST, aspartate aminotransferase; BchE, butyrylcholinesterase; 2,4-D, 2,4-dichlorophenoxyacetic acid; 2,4-DCP, 2,4-dichlorophenol; AChE, acetylcholinesterase; DDT, 1,1,1-trichloro-2,2-bis(4-chlorophenyl)ethane; DTC, dithiocarbamates; GPx, glutathione peroxidase; GSH, GSSG, reduced and oxidized glutathione; GST, glutathione-S-transferases; LDH, lactate dehydrogenase; NF-kB, transcription factor nuclear factor kappa B; OP, organophosphorous pesticide; ROS, reactive oxygen species; SOD, superoxide dismutase.

## Introduction

Pesticides are synthesized substances or biological agents used for attracting, seducing, destroying, or mitigating any pest. They are mainly applied in agriculture to protect crops from insects, weeds, and bacterial or fungal diseases during growth and to protect foods during storage from rats, mice, insects or diverse biological contaminants (Bolognesi and Merlo, 2011[15]). Some pesticides, like herbicides, are applied to clear roadside weeds, trees, and shrubs and are commonly applied in ponds and lakes to control unwanted aquatic plants. Others are used to kill or inhibit growth of fungi or insects that parasitize crops (Gupta, 2011[68]). Thus, being a heterogeneous category, pesticides occupy a unique position among synthetic chemicals that humans encounter daily. They can now be found almost everywhere worldwide. Pesticides originating from human activity can also enter water bodies through surface runoff, leaching, and/or erosion (Khan and Law, 2005[91]). Meanwhile, drift, evaporation, and wind erosion can carry pesticide residues into the atmosphere, which can lead to contamination of surface waters, soils, flora, and fauna via precipitation, often at sites distant from their place of origin (Dubus et al., 2000[43]). 

Pesticides are characterized by various degrees of toxicity to target and non-target organisms (Bolognesi and Merlo, 2011[15]; Khan and Law, 2005[91]). Because of cumulative properties of many pesticides (Wilkinson et al., 2000[189]), they circulate in ecosystems and may be accumulated by many living organisms and even migrate through food chains. To recognize herbicide impact some biological subjects, individuals, species, or communities, are preferentially used as models for evaluation of hazardous influences. Pesticides may enter the body by different ways depending on species, metabolic peculiarities, and susceptibility to toxins (Hodgson, 2010[73]). However, if a chemical already entered an organism, the organism must be able to deal with it in order to neutralize or minimize its deleterious effects via biotransformation, conjugation, isolation and/or excretion into the environment or via a combination of these mechanisms. All these efforts are directed to prevent or minimize damage to the organism. Elimination of pesticides can be implemented in at least two ways: either by excretion in their original form or after biotransformation and/or conjugation with different compounds by the organism (van der Oost et al., 2003[180]). Interestingly, sometimes biotransformation can result in more hazardous products than the initial pesticide. Processing of pesticides depending on their properties, dose, and routes of entry can substantially affect the organism. For example, pesticides can cause endocrine disruptions and neurological disturbancіes, influence immune system, reproduction, development (Khan and Law, 2005[91]). In view of this, the toxicity of pesticide exposure to non-target organisms is a substantial concern around the world.

The mode of action of numerous pesticides is diverse and often cannot be specifically classified. It is well known that organophosphorus pesticides are extremely neurotoxic since they irreversibly inhibit acetylcholinesterase, an enzyme that hydrolyzes the neurotransmitter acetylcholine at neuromuscular junctions and brain cholinergic synapses (Galloway and Handy, 2003[57]; van der Oost et al., 2003[180]). Many dithiocarbamates (DTC) induce intraneuronal oxidative stress leading to neuronal damage because the metal ions released during their biotransformation can enhance the steady-state levels of reactive oxygen species (ROS) and stimulate ROS-induced oxidation of lipids and proteins, or inactivate certain enzymes resulting in neurotoxic effects (Fitsanakis et al., 2002[53]; Nobel et al., 1995[132]). A number of pesticides cause endocrine disruption by interfering with the production, release, transport, metabolism, action, or elimination of hormones (Bolognesi and Merlo, 2011[15]; Khan and Law, 2005[91]). Pesticides also may increase steady-state ROS levels, stimulate ROS-induced modification of cellular components, affect core homeostatic and regulatory processes, or deplete antioxidant defenses that collectively result in the development of oxidative stress (Abdollahi et al., 2004[1]; Banerjee et al., 2001[8]; Lushchak, 2011[107][109]). Since ROS interact with DNA in different ways, an increase in their steady-state levels can enhance the chance to interact with the genetic material and cause genotoxicity leading to diverse mutations (Franco et al., 2010[54]).

This review considers general mechanisms of pesticide-promoted toxicity in target and non-target organisms. Because pesticides are present and circulate everywhere in the environment, we have analyzed the main routes of penetration and processing of these chemicals in nature as well as the peculiarities of their metabolism in living organisms. 

## Persistent Status of Pesticides in the Environment

From the viewpoint of environmental protection, ecotoxicological studies of the natural environment have become very important in recent decades since pesticides regularly enter the environment. They may disturb the natural balance of the ecosystem and cause substantial ecological changes even if used according to good agricultural practices. Whereas problems arising from pesticide use are most often linked with agriculture or forestry practices, they are also present as a common component of urban wastewater accumulating as the result of weed treatment along roads or rail lines, as well as from gardens, parks, and urban woodland areas. These include pesticides of the triazine group, the phenyl urea group (e.g. chlorotoluron, isoproturon and diuron), the phenoxy acid group (e.g. 2,4-dichlorophenoxyacetic acid (2,4-D)), etc. (Revitt et al., 1999[142]) (for details see section “CLASSIFICATION OF PESTICIDES: GENERAL APPRAISAL”). The ability of various pesticides to affect organisms in different ways complicates the risk assessment based on the environmental levels. Deleterious effects are often difficult to detect in targeted organisms since many of these effects tend to manifest only after prolonged exposure. When the effects finally become obvious, destructive processes may already be irreversible (van der Oost et al., 2003[180]). Furthermore, due to the long-term persistence of many pesticides (or products of their conversion) in the environment, resulting from non-controlled or poorly controlled use in agriculture and other human activities, ecosystems may be substantially modified. Because of their cumulative properties, pesticides circulate and become accumulated in many living organisms some of which are used as model subjects for investigation of their hazardous effects. 

### Pesticides and their circulation in the biosphere

**Figure 1:**
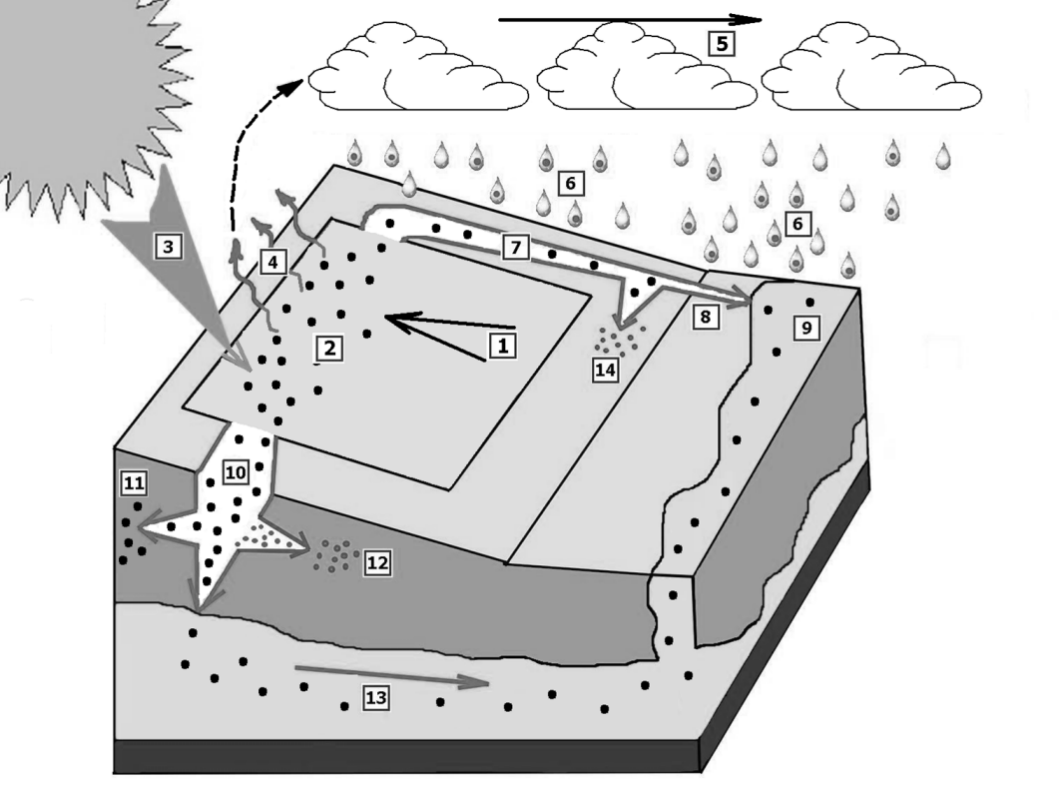
The movement of pesticide in the hydrologic cycle. Diffuse water pollution through pesticides occurs either due to evaporation (4) with short and long-distance transfer (5), surface runoff (8) or leaching to groundwater (13). 1 - pesticide application; 2 - absorbed by crop; 3 - degraded by ultraviolet light; 4 - evaporation (vaporized to atmosphere); 5 - short and long-distance transport; 6 - deposited by rainfall; 7 - runoff; 8 - surface runoff to lakes and rivers; 9 - polluted waters; 10 - seepage; 11 - adheres to soil particles; 12 - biodegradation (degraded by bacterial oxidation or chemical hydrolysis); 13 - leaching (groundwater discharge to streams); 14 - pollution of surrounding territory.

Pesticides can be found almost everywhere worldwide. Large numbers of pesticides can persist in water bodies, air, fog, rain, and soils (Bolognesi and Merlo, 2011[15]). The fate of a contaminant in the environment is affected by a variety of physical, chemical, and biological processes that can affect their processing as well as their interactions with environmental components. Pesticides most commonly enter bodies of water due to runoff from adjacent fields and roads (Figure 1(Fig. 1)). Other routes include direct spray, airborne drift, intentional dumping, improper mixing, and contaminated groundwater. Pesticide penetration into groundwater is controlled by two factors: water applied and interaction with organisms and solid particles, i.e. a balance between absorption and adsorption (Huggenberger et al., 1973[77]). 

Eventually, when chemicals enter ecosystems, transformation occurs in various ways depending upon their physical and chemical properties and interaction with other environmental components. For example, water solubility is a key characteristic of a chemical but is affected by several parameters including temperature, pH, salinity, turbidity, and the presence of other chemicals in the microenvironment (Rand et al., 1995[140]). Highly water-soluble pesticides, such as 2,4-D, are less persistent in the environment, and are most likely to biodegrade quickly. Because of this, they are not likely to be accumulated in the soil or sediments, volatilize, or bioconcentrate in organisms. Hydrolysis is a common way to degrade many pesticides, particularly those chemicals that possess chemical bonds that are potentially hydrolyzable at environmental pH (Katagi, 2010[88]). Some other contaminants have chemical structures that can be decomposed by visible or UV light in a process called photolysis. If the chemical possesses double bonds between carbon atoms or other chemical elements, and absorbs light at visible or UV wavelengths, it can potentially undergo direct photolysis (Hemond and Fechner, 1994[70]; Sparks and Nauen, 2015[162]). Non-absorbing compounds may undergo indirect photolysis, where light-absorbing molecules commonly persisting in water absorb photons and subsequently transfer their energy to non-absorbing compounds. Indirect photolysis can also occur when transient oxidants such as hydroxyl radicals or singlet oxygen attack pesticide molecules (Hemond and Fechner, 1994[70]; Sparks and Nauen, 2015[162]). 

Environmental temperature also plays a significant role since temperature determines not only the level of dissolved oxygen in the water, but can also affect the behavior of diverse chemicals in an aqueous environment by influencing solubility, volatility, and chemical activity of pesticides (Chovanec et al., 2003[29]). The bioaccumulation and toxicity of chemicals are also influenced by temperature. Increased contact time between the body surface of an organism and a pesticide will intensify bioconcentration, a form of bioaccumulation in which the pesticide is accumulated directly from the environment. In water, pesticides may act either alone, or in concert with many biological, physical, or other chemical factors that can affect aquatic organisms. Thus, it is not a simple matter to determine the mechanisms of pesticide toxicity, which may be further complicated by environmental factors such as elevated temperature, low dissolved oxygen levels, or by bacterial infections and parasite invasions (Khan and Law, 2005[91]). Since the danger of pesticide contamination is high, and many other factors can act individually or in combination to produce health harm, a clear protocol needs to be established to resolve these issues.

Historically, chemical exposure in the workplace has been assessed through environmental monitoring. Analytical procedures for the detection of pesticides and their metabolites in biological samples (blood, urine, saliva, sweat, leaves, roots, etc.) have been developed to study patterns of absorption, transformation, and excretion of these compounds (Bolognesi and Merlo, 2011[15]). Acute effects of pesticides have been adequately evaluated in different test/model organisms (Atamaniuk et al., 2013[3]; El-Sayed et al., 2007[49]; Prusty et al., 2011[139]). Although acute responses to exposure are well known for many pesticides, human data on their delayed effects are much more limited since human exposure to pesticides is extremely complex as a result of occupational or environmental influences.

### Transfer and bioaccumulation of pesticides in the food chains

Pesticides are known to be widespread environmental pollutants due to their bioaccumulation and persistence in the ecosystems. Residues of these compounds have been detected in different biological media of test organisms (Bolognesi and Merlo, 2011[15]). Because most organisms interact with each other in the food web, knowledge about pesticide migration and bioconcentration from dietary exposure is important for the evaluation of their real environmental effects (Katagi, 2010[88]). Runoff and erosion can be major routes of chemical entry into surface waters (Giddings et al., 2005[61]) and so, for aquatic organisms, persistent chemicals may also accumulate through other mechanisms including via the direct uptake from water by gills or skin (bioconcentration), via uptake of suspended particles (ingestion), and via the consumption of contaminated food (biomagnification) (van der Oost et al., 2003[180]). Terrestrial wildlife can be exposed to pesticides via consumption of contaminated food or water (Solomon et al., 2008[160]). 

**Figure 2:**
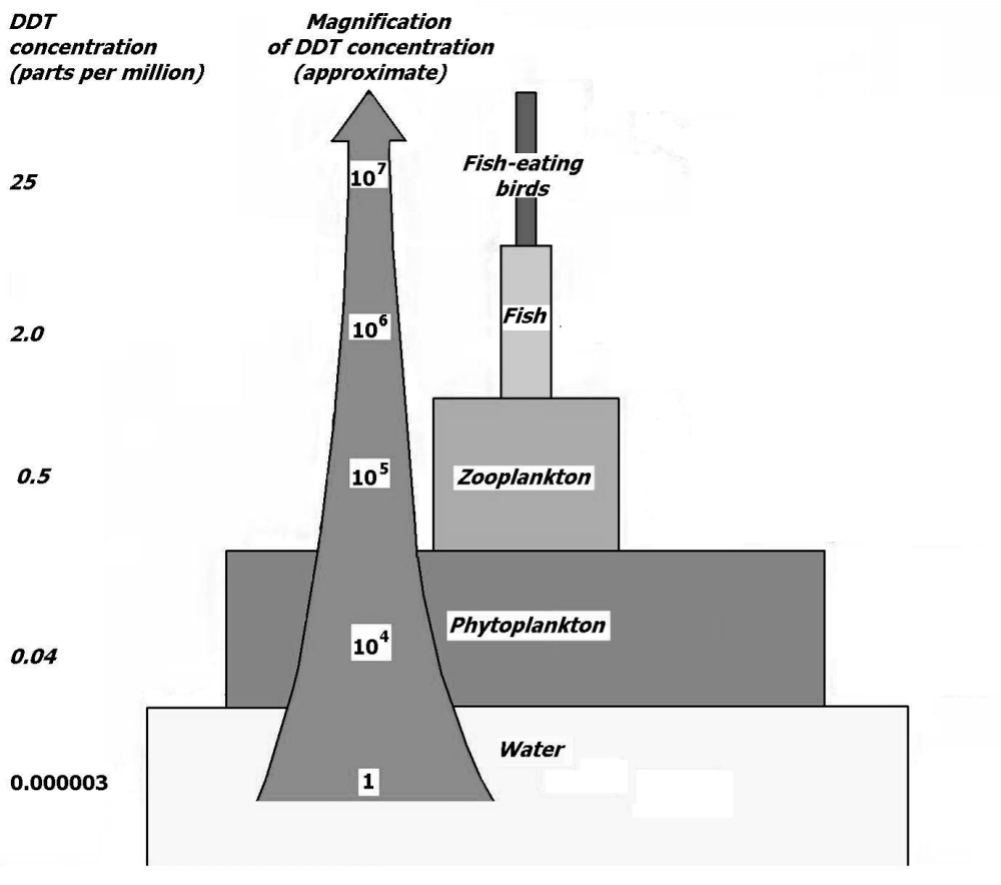
Bioaccumulation of DDT in the food chain. Each successive consumer in the food chain accumulates contaminants to a higher level, thus magnifying the exposure when moving up the food chain

The term “bioconcentration” is broadly used to describe the process of pesticide entrance into organisms. Katagi (2010[88]) disсussed three main factors determining bioconcentration processes: (*i*) physicochemical properties of the individual chemicals, (*ii*) physiological disposition of the organism penetrated, and (*iii*) surrounding environmental conditions. Since biological membranes are the primary barriers for chemicals, the physicochemical properties of pesticides such as steric parameters (e.g. molecular size and shape) and water or lipid solubility are critically important (Landrum and Fisher, 1998[97]). Among the range of physiological properties that exist in organisms, lipid content is considered to be the most important determinant for pesticide bioconcentration because lipid-soluble pesticides are especially prone to bioaccumulation. Lipid influence on pesticide intake may be followed by metabolism or excretion, which are substantially affected by the physiological state of organisms (Katagi, 2010[88]). Finally, the rate of bioconcentration also depends to some extent on environmental conditions. Hence, for chemicals having a dissociable functional group, bioconcentration may be affected by the environmental pH value. The hardness of water was also reported to affect both uptake and elimination processes of pesticides (Kawatski and Bittner, 1975[89]). In the aquatic environment, the presence of bottom sediments also substantially complicates an investigation of bioconcentration. Aquatic organisms usually ingest prey, sediment particles, and detritus contaminated by chemicals, and this may affect bioaccumulation rates (Katagi, 2010[88]). Therefore, the bioconcentration of pesticides often leads to their bioaccumulation that includes added effects of dietary uptake through food consumption and intake of sediments (Miyamoto et al., 1990[122]). Figure 2(Fig. 2) shows how DDT [1,1,1-trichloro-2,2-bis(4-chlorophenyl)ethane] becomes concentrated in the tissues of organisms. 

Concentration of DDT in living organisms results from imbalance between its absorption, metabolization, and excretion. Thus, when a pesticide enters a water source, it first accumulates in and contaminates plankton. When small fish species eat plankton, they are then contaminated and when bigger fish eat smaller ones, they are also contaminated. Such events lead to DDT accumulation through food chains and its persistence in these chains. Hence, food is the most significant source of toxicants that bioaccumulate along food chains. It is commonly accepted that if the levels of pesticides persisting in the organism are enhanced through two or more trophic levels in a food web, that the process is referred to as “biomagnification” (Connell et al., 1988[32]). There are two different mechanisms providing biomagnification: active and passive transportation. In the first case, specific systems are responsible for pesticide entrance.

In the second case, however, biomagnification can be related to some driving force for net passive chemical transport; i.e. every penetrating organic chemical has a particular chemical activity or chemical potential which promotes the tendency of the chemical to be released from a phase when driving on the food chain (Gobas et al., 1988[62]). Biomagnification also can be determined as the ratio between the uptake of chemicals from food and their clearance (Sijm et al., 1992[158]).

### Uptake and bioprocessing of pesticides

Pesticides may enter organisms in different ways. Due to differences in metabolism and other characteristics, species, strains, and individuals may vary greatly in their susceptibility to pesticides. Aquatic organisms may absorb dissolved chemicals directly from the water across respiratory organs (e.g., gills), the body surface, or via intake of contaminated food, suspended particles or sediments (Katagi, 2010[88]; Lushchak, 2011[109]). Most terrestrial animals also absorb pesticides through skin, respiratory and/or gastrointestinal tract surfaces. The skin and nasal mucosa are the main portals of entry for different pesticides (Hodgson, 2010[73]). A few pesticides are known to give rise to toxic endpoints in the nasal tissues; some of them have been identified to cause nasal lesions or tumors in experimental animals (Hodgson, 2010[73]). The lung is also a primary site of exposure to airborne environmental pollutants closely contacting with blood (Ding and Kaminsky, 2003[40]). 

Pesticide acquisition from all routes of exposure eventually comes to the liver for disposition, liver being the primary site of pesticide biotransformation for facilitated clearance through excretion of water-soluble products of detoxification. However, the high level of oxidative metabolism in liver also makes it a possible target for more toxic metabolic products appearing due to biotransformation of certain xenobiotics (Hodgson and Goldstein, 2001[74]). For example, pesticide poisoning accompanied by acute liver intoxication has been associated with chronic pesticide exposure (Hodgson and Goldstein, 2001[74]). Kidney is a secondary organ involved in detoxification related to big extent by its high blood flow and its ability to concentrate and convert pesticides due to which it is a target for xenobiotic toxicity (Husak et al., 2014[79], 2017[80]; Husak, 2015[78]). Very little is known about xenobiotic detoxification in the central nervous system although several studies have demonstrated efficient relationships between development of neurotoxicity and exposure to organophosphorus compounds (Galloway and Handy, 2003[57]; Vani et al., 2011[181]). 

**Figure 3:**
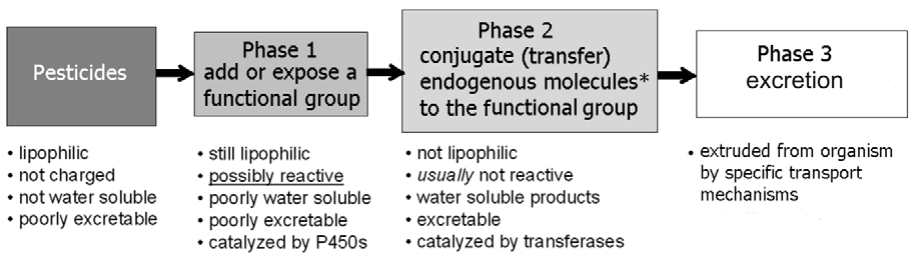
Biotransformation of pesticides. Description in the text. *glutathione, carbohydrates, amino acids, sulfates, acetyl groups

Biotransformation is one of the most important factors governing bioconcentration, bioaccumulation, and detoxification of pesticides (Katagi, 2010[88]). Williams (1959[190]) first suggested that the metabolism of xenobiotics generally occurs in two stages that are now generally classified as phase I and phase II detoxification reactions that proceed successively to facilitate elimination of pesticides (Hodgson, 2010[73]; Katagi, 2010[88]). Phase I stage involves predominantly oxidation, reduction, and hydrolysis and serves to introduce a polar group into hydrophobic molecules, i.e. produce derivatives containing -OH, -COOH, -NH2, and -SH functional groups (Figure 3(Fig. 3)). Such oxidation is usually catalyzed by mixed function oxidases, including cytochrome P450 enzymes and has been extensively investigated (Watanabe, 2000[187]). Located in endoplasmic reticulum P450 enzymes usually function as terminal oxidases of electron-transport chains. Phenols are thought to be primarily oxidized by monooxygenases to the corresponding catechol derivatives followed by ring cleavage by 2,3-dioxygenases (Semple et al., 1999[154]). Lipoxygenases, dioxygenate mainly polyenoic fatty acids, but also take part in conversion of different xenobiotics primary via a direct hydrogen abstraction in the reactions oxidation, epoxidation, hydroxylation, sulfoxidation, desulfuration, dearylation, and N-dealkylation as well as are capable of glutathione conjugation of certain xenobiotics (Kulkarni, 2001[95]). Reductive dehalogenation and dehydrohalogenation, typical reactions for biotransformation of DDT, as well as reduction of nitro- and S-oxide groups were also described. Hydrolysis, catalyzed by various esterases, is common in the metabolism of organophosphorus and pyrethroid pesticides (Katagi, 2010[88]; Mangas et al., 2017[116]). Based on reaction profiles, esterases are classified functionally into three categories (Thompson, 1999[172]; Wheelock et al., 2005[188]):

1. A-esterases that include phosphotriester hydrolases that hydrolyze organophosphorus (OP) compounds and are not inhibited by OPs.
2. B-esterases including cholinesterases and carboxyesterases that are typically inhibited by OPs as a result of the extremely slow dephosphorylation of tetrahedral intermediates formed between OPs and a serine residue at their active sites (Fukuto, 1990[55]). Carboxyesterases are well known to hydrolyze pyrethroids and carbamates. Among cholinesterases, acetylcholine (AchE) and butyrylcholine (BchE) esterases have been found in neuromuscular junctions, whereas carboxyesterases are usually distributed in all tissues and hydrolyze a wide variety of endogeneous and exogeneous esters (Galloway et al., 2002[58]).
3. C-esterases that include acetylesterases not inhibited by OPs.

The phase II detoxification system, consisting primarily products of conjugation reactions, includes the combination of the products of phase I reactions with carbohydrates, reduced glutathione (GSH), sulfate, or amino acids to form water-soluble excretable products (Figure 3(Fig. 3)) (Lushchak, 2011[109]). Acetylation, formylation, and conjugation with amino acids are mostly used for amino and carboxyl groups after reduction of the nitro group or ester bond cleavage. Glucose conjugates can be further metabolized by acetylation or conjugation with malonic acid or carbohydrates (Katagi, 2010[88]). Glutathione-S-transferases (GSTs) are widely distributed in terrestrial and aquatic organisms and these enzymes catalyze the transfer of tripeptide GSH to electrophilic chemicals such as epoxides, halides, and arene oxides that are formed by phase I oxidation (James, 1994[83]; Lushchak, 2012[112]). The conjugates formed then undergo further metabolism via catalysis by peptidases and N-acetyltransferase via two intermediates and finally to conjugates of mercapturic acid. Many chemicals (e.g. chlorophenol derivatives) are known to inhibit a GST (LeBlanc and Cochrane, 1987[98]). Such metabolic profiles may be common in all eukaryotic organisms, but the contribution of these reactions depends on species.

Reactions of phase II detoxification are not the final stage of the overall process. The xenobiotic conjugates can be metabolized, for example with glutathione, and excreted from the living organisms (Lushchak, 2012[112]). The system responsible for excretion of transformed and original pesticides has been called phase III detoxification (Figure 3(Fig. 3)). Specific ATP-binding cassette transporters provide the ATP-dependent excretion of diverse hydrophilic anions to the extracellular medium (Homolya et al., 2003[75]; Nies and Keppler, 2007[129]). 

Organisms eliminate absorbed chemicals in two forms: they are either excreted in original form (the parent compound) or as products of their biotransformation. The products of biotransformation generally are more hydrophilic compounds and are more readily excreted than parental ones (Vermeulen, 1996[183]). In animals, liver is the organ most commonly involved in biotransformation of foreign compounds due to its function, position among other organs and extensive blood supply. Biotransformation usually alters the toxicity of compounds making them either more or less toxic to the organism than the initial compound (van der Oost et al., 2003[180]). The skin also contains many xenobiotic metabolizing enzymes and some are inducible, primarily by polycyclic hydrocarbons (Baron et al., 2008[10]). Because of kidney role in the organism related with high blood flow and presence of renal xenobiotic metabolizing systems, it is also the target for xenobiotic toxicity (Speerschneider and Dekant, 1995[163]). Interestingly, among animals, we know that the capacity for biotransformation and elimination of xenobiotics is often positively correlated with an organism's capability to survive general stress conditions. Usually, more stress-tolerant organisms demonstrate lower sensitivity to xenobiotics (Banaszkiewicz, 2010[6]). 

Plants possess coordinated defense mechanisms to natural and synthetic toxicants (Zhang et al., 2007[194]). Similarly to animals, plants possess systems of biotransformation to cope with xenobiotics. Hence, the capacity of plants to detoxify herbicides metabolically via complex multistep processes clearly demonstrates their highly specific defense systems that also show extraordinary diversity among species (Kreuz et al., 1996[92]). In modern agriculture, selective herbicides are widely used that are safe for use on particular crops, but can efficiently control associated weeds (Riechers et al., 2010[143]). They frequently are claimed to be low toxic for non targeted organisms.

In plants, several groups of enzymes are used for herbicide detoxification along with transporters providing release of pesticides in environment (Bounds and Hutson, 2000[18]; Kreuz et al., 1996[92]). As in animals, plants possess three defense systems or phases of detoxification. Phase I reactions involve oxidation by P450 cytochromes and hydrolysis by carboxylesterases. Phase II includes conjugation of original or transformed xenobiotics with endogenous molecules such as GSH in the reactions catalyzed by GSTs, glucuronic acid in reactions involving UDP-glucuronosyltransferase, or sulfate in reactions conducted by sulfotransferases. In phase III biotransformed xenobiotics alone or in conjugated form are transported into the vacuole or extruded from the plant by specific transport mechanisms. Interestingly, plants are also capable of further processing of conjugates such as by partial degradation, secondary conjugations, or incorporation into cell wall constituents (sometimes called phase IV detoxification) (Riechers et al., 2010[143]).

## Classification of Pesticides: General Appraisal

The term ”*pesticide*” indicates any substance or mixture of substances used to kill, repel, or otherwise control a ”*pest*”, including insects, snails, rodents, fungi, bacteria, and weeds (Bolognesi and Merlo, 2011[15]). The “green revolution” caused rapid growth in the application of pesticides which contributed significantly to increased production and expansion of the range of pesticide products. In this regard, there is an urgent need to develop a classification of pesticides that would provide essential clues to navigate the mass of existing compounds and choose the best one for the target application. When compiling the classification of pesticides it is very difficult to meet one single principle, so in most cases, combined approaches are preferred. There are three general characteristics according to which pesticides may be classified: 

(A) **assignment** (or type of pest, target group) - e.g., herbicides, fungicides etc;

(B) **method of pesticide impact** - *contact* (in some cases acting externally to dry the body of the pest or to create a gas-tight film that blocks normal gas exchange, or in other cases penetrating through the integument to strike the nervous system, etc.), *systemic* (pesticides easily penetrate the organism barriers and affect all organs), *fumigants* - chemical compounds that enter the body through inhalation to affect bloodstream, enzymes and nervous systems of living organisms, and complex preparations; 

(C) **chemical nature of the pesticide** - is the most specific way to differentiate the multiple classes and subclasses of compounds that exhibit a vast array of chemically diverse structures (Franco et al., 2010[54]), as detailed in the Pesticide Manual published by British Crop Protection Council (Tomlin, 2000[174]). From this, depending on chemical structure, the most popular pesticides may be divided into the following groups (Bolognesi and Merlo, 2011[15]; Franco et al., 2010[54]; Katagi, 2010[88]):

1. Organochlorines (e.g., endosulfan, hexachlorobenzene);
2. Organophosphates (e.g., diazinon, omethoate, glyphosate);
3. Carbamic and thiocarbamide derivatives (e.g., aldicarb, carbofuran, oxamyl, carbaryl);
4. Carboxylic acids and their derivatives (e.g., pentanal, butanamide, butanamide);
5. Urea derivatives (e.g., fenuron, metoxuron, diuron, linuron, monuron);
6. Heterocyclic compounds (e.g., benzimidazole, triazole derivatives);
7. Phenol and nitrophenol derivatives (e.g., dinocap, dinoseb);
8. Hydrocarbons, ketones, aldehydes and their derivatives (e.g., benzene, toluene, cerenox);
9. Fluorine-containing compounds (e.g., cryolite, acetoprole, dichlofluanid);
10. Copper-containing compounds (e.g., champion WP, caocobre, macc 80);
11. Metal-organic compounds (e.g., mancozeb, maneb, zineb, nabam);
12. Synthetic pyrethroids and others (e.g., allethrin, cypermethrin, fluvalinate).

There are also other approaches that may serve as important tools used for pesticide classification. For example, their toxicity is of great interest to modern science. However, this parameter is too changeable to become a classification mechanism for pesticides. Toxicity of pesticides depends on temperature, dose, permeation rate, degradation time etc., usually with broad fluctuations that makes it difficult to use as a classification parameter. 

Selectivity is known to be among the most desired properties of pesticides, i.e. ideally pesticides should act specifically against certain target organisms without severely affecting others (Bolognesi and Merlo, 2011[15]). Theoretically, pesticide chemicals might be designed or selected that uniquely attack a functional system or target molecules peculiar to the ”pest” with either absent or less critical in its effects on other organisms. For example, chitin synthetase inhibitors are selectively toxic to invertebrates with exoskeleton (Bolognesi and Merlo, 2011[15]). Interestingly, the same approach can be used to treat fungi that also possess chitin. Such inhibitors can also potentially serve as fungicides, but the information on such application is very old and scarce (Leighton et al., 1981[99]). Therefore, one may expect that inhibitors of chitin synthetase may affect both insects and fungi. 

### Herbicides and their mode of action

*Herbicides*, or chemical weed killers, provide an effective and economical means of weed control. The worldwide use of herbicides accounts for almost 48 % of the total pesticide usage. In the last three decades, herbicides have represented the most rapidly growing segment of the pesticide industry (Gupta, 2011[68]). Similar to other pesticides, herbicides may be classified according to specificity, chemical nature, time of application, and mode of action (Peterson et al., 2013[137]). Improper use of herbicides has resulted in human health problems and the mechanisms of toxicity of many pesticides to non-target organisms remain poorly studied. Research into understanding the mode of action of herbicides may be an important tool to improve their efficiency, application methods in various agricultural practices, handle weed resistance problems, and explore toxic properties (Jablonkai, 2011[82]).

**Figure 4:**
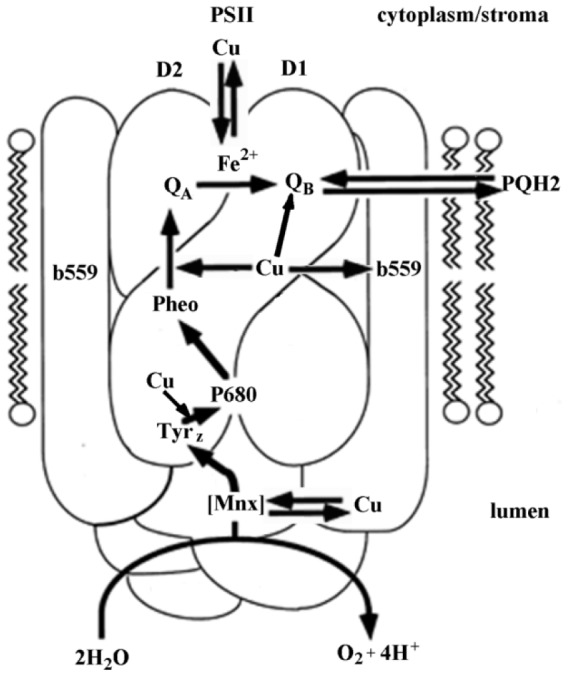
Cu-inhibitory sites and active sites of different electron donors and acceptors in PSII-mediated electron transport. PSII, photosystem II; D1 and D2, bind the electron carriers involved in transfer of electrons from Tyr_z_ to plastoquinone; b559, cytochrome b559; Tyr_z_, tyrosine residue active electron transfer from the manganese complex to reaction centre P680; Pheo, pheophytin; Q_A_ and Q_B_, bound plastoquinone; P680, reaction center of chlorophyll (primary electron donor); PQ, reduced plastoquinone (Husak, 2015).

Since most herbicides are synthesized to target specific plant metabolic pathways (e.g. photosynthesis, plant hormone action, regulation of cell division, etc.), they kill plants in different ways (Bolognesi and Merlo, 2011[15]; Peterson et al., 2013[137]). However, before killing the target, the herbicide must contact the site of action in the weed otherwise its actions are useless. Herbicides can affect various sites in plants and at the site of action each herbicide manifests different mechanisms, which are grouped as follows (Peterson et al., 2013[137]):

1. **Growth Regulators.** This type of herbicides is used to control broadleaf weeds. They influence plants stimulating their growth like natural hormones shifting in this manner hormone balance. For example, 2,4-dichlorophenoxiacetic acid belongs to this group. The mechanism found for herbicidal activity of 2,4-D is based on its auxin-like capacity. A receptor for auxin was reported to recognize synthetic auxin analogues such as 2,4-D (Jablonkai, 2011[82]).
2. **Seedling Growth Inhibitors.** Among these herbicides, thiocarbamates and acid amides act as powerful shoot and root growth inhibitors. These herbicides appear to interfere with normal plant growth, especially at growth points. The herbicides that inhibit cell division also belong to this category. They are frequently mitotic poisons and are represented mostly by dinitroanilines.
3. **Photosynthesis Inhibitors.** These herbicides (e.g. triazines, copper-containing pesticides) block photosynthesis via disruption of biomembranes by highly active molecules. The susceptible plants die from a buildup of highly reactive molecules that destroy cell membranes. Triazine herbicides (including atrazine and simazine) are effective and inexpensive herbicides used to control a wide spectrum of broadleaf weeds and selective grasses (Sathiakumar et al., 2011[148]). For example, atrazine inhibits photosynthesis via competition with plastoquinone II at its binding site and blocks electron transport in photosystem II (Devine et al., 1993[39]). This inhibition results in the cessation of carbohydrate synthesis, leading to a subsequent reduction in the carbon pool and a buildup of CO_2_ within the plant cell (Giddings et al., 2005[61]). At high concentrations, copper or copper-containing pesticides can interrupt electron transport through photosystem II. Jegerschöld et al. (1995[85]) demonstrated that copper ions blocked the electron donation from Tyrz to P680 (Figure 4(Fig. 4); Reference in Figure 4: Husak, 2015[78]). Moreoever, the central magnesium atom of chlorophyll was found to be substituted by ions of mercury, copper, or cadmium, inhibiting in this manner operation of photosystem (Küpper et al., 1996[96]). Copper ions were found to oxidize the low potential form cyt *b*_559_ at low concentrations (1-10 µM) and the high potential form at higher concentrations (10-100 µM), probably by deprotonation of this labile cyt *b*_559_ form (Burda et al., 2003[21]).
4. **Inhibitors of Amino Acid Biosynthesis.** These herbicides block biosynthesis of certain amino acids. For example, glyphosate [N-(phosphonomethyl)glycine], an active ingredient of herbicide Roundup, inhibits plant biosynthesis of the aromatic amino acids such as tyrosine, tryptophan, and phenylalanine. There are some other targets for these chemicals. Thus, several classes of herbicides may inhibit acetohydroxyacid synthase, which catalyzes the first common step in the biosynthesis of valine, leucine, and isoleucine, or 4-hydroxyphenylpyruvate dioxygenase, a key enzyme in tyrosine catabolism and carotenoid synthesis (Duggleby et al., 2008[46]; Garcia et al., 2017[59]). Several compounds are potent inhibitors of glutamine synthase that catalyzes incorporation of ammonia onto glutamate (Jablonkai, 2011[82]; Tarazona et al., 2017[171]).
5. **Lipid Biosynthesis Inhibitors.** Herbicides of this group such as fluazifop, sethoxydim, are used mainly for postemergent grass suppression. They inhibit biosynthesis of lipids and it results, particularly, in impossibility to form biological membranes.
6. **Cell Membrane Disrupters.** These chemicals are light-activated postemergence contact herbicides. Injury symptoms are represented by browning (necrosis) of the tissue appear first as water soaked foliage. Paraquat and diquat are the most typical representatives of this group.
7. **Pigment Biosynthesis Inhibitors.** These herbicides (e.g. clomazone) inhibit biosynthesis of photosynthetic pigments called carotenoids, which protect chlorophyll from destruction by light. Without carotenoids, chlorophyll is destroyed and the plants are unable to carry out photosynthesis.

### Fungicides and their mode of action

**Figure 5:**
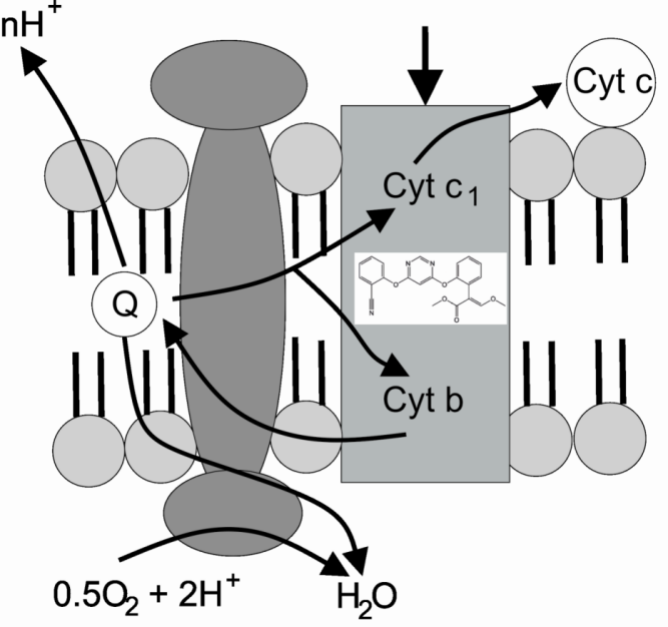
Quinone/quinol (Q) site of electron transfer and azoxystrobin inhibition. Description in the text (Modified from Casida and Durkin (2017)).

*Fungicides* are agents that kill, repel, prevent, or otherwise mitigate fungi and they are used to protect tubers, fruits, and vegetables during storage and plant growth (Gupta, 2011[68]). The mode of action of fungicides depends on their protection role in plants. Thus, there are preventive fungicides that prevent infections, antisporulants that prevent spore production, and curative fungicides that inhibit the development of a disease following infection (Bolognesi and Merlo, 2011[15]). Moreover, some fungicides are single-site active ones and affect a fungus or a single critical enzyme or protein critically needed by fungus, whereas others are multisite ones that deal with different metabolic sites within the fungus (Bolognesi and Merlo, 2011[15]). Similar to herbicides, the mode of action of fungicides is closely related to specific fungal metabolic pathways, but this task is more difficult due to certain similarities between fungi and animals. Nevertheless, a few general mechanisms of fungicide activity can be defined:

1. **Ergosterol Biosynthesis Blockers.** Conazoles possess the ability to block the synthesis of ergosterol that is an essential component of the fungal cell membrane. These fungicides primarily inhibit the cytochrome P450 (CYP-51) or lanosterol-14α-demethylase, the only members of the cytochrome family that are found in animals, plants, fungi, and prokaryotes. Conazoles have a broad antifungal activity and are used as pharmaceuticals to treat topical and systemic fungal infections (Bolognesi and Merlo, 2011[15]).
2. **Protein Biosynthesis Inhibitors.** Dithianon acts as a multisite inhibitor of protein formation modifying the sulfhydryl groups of many proteins. This protein synthesis inhibition prevents spore germination and germ tube growth. Benzimidazoles, for example, suppress the reassembly of depolymerized spindle microtubule division. Although these compounds exhibit specific efficiency against fungal organisms, they also target mammalian microtubule assembly dynamics (Bolognesi and Merlo, 2011[15]; Oruc, 2010[133]).
3. **Inhibitors of mitochondrial respiration.** Azoxystrobin inhibits mitochondrial respiration and energy production by blocking electron transfer at the quinone “outside” site of the cytochrome *bc_1_* complex between cytochrome *b* and cytochrome *c_1_* referred to as the ubiquinol oxidizing or Q_o_ site and thereby ultimately prevent the generation of ATP (Figure 5(Fig. 5); Reference in Figure 5: Casida and Durkin, 2017[25]) (Balba, 2007[4]; Casida and Durkin, 2017[25]).
4. **Multisite Fungicides.** The widespread dithiocarbamate fungicides (mancozeb, zineb) are nonspecific and affect different biochemical processes in target fungi. These include inhibition of antioxidant enzymes to disturb redox balance in cells (Lushchak et al., 2005[106]), suppression of respiration, and some of them also inhibit the nuclear factor-kB (NF-kB) signaling cascade (Rath et al., 2011[141]).

Chemical classes of fungicides include (Balba, 2007[4]):

- Benzimidazoles - benomyl, thiophanate-methyl.
- Carbamic acid derivates, namely dithiocarbamates and ethylene(bis)dithiocarbamates.
- Halogenated substituted monocyclic aromatics (substituted benzenes) such as chlorothalonil.
- Organomercury compounds. They interact with sulfhydryl groups in proteins. In animals, they may block transfer of amino acids across the blood-brain barrier and interfere with protein biosynthesis.
- Phthalimides or chloroalkylthiodicarboximides are chemicals with broad-spectrum fungicidal effects (captan, folpet, captafol, etc.) used as surface protectants and are usually believed to be nontoxic for mammals.

### Insecticides and their mode of action 

**Figure 6:**
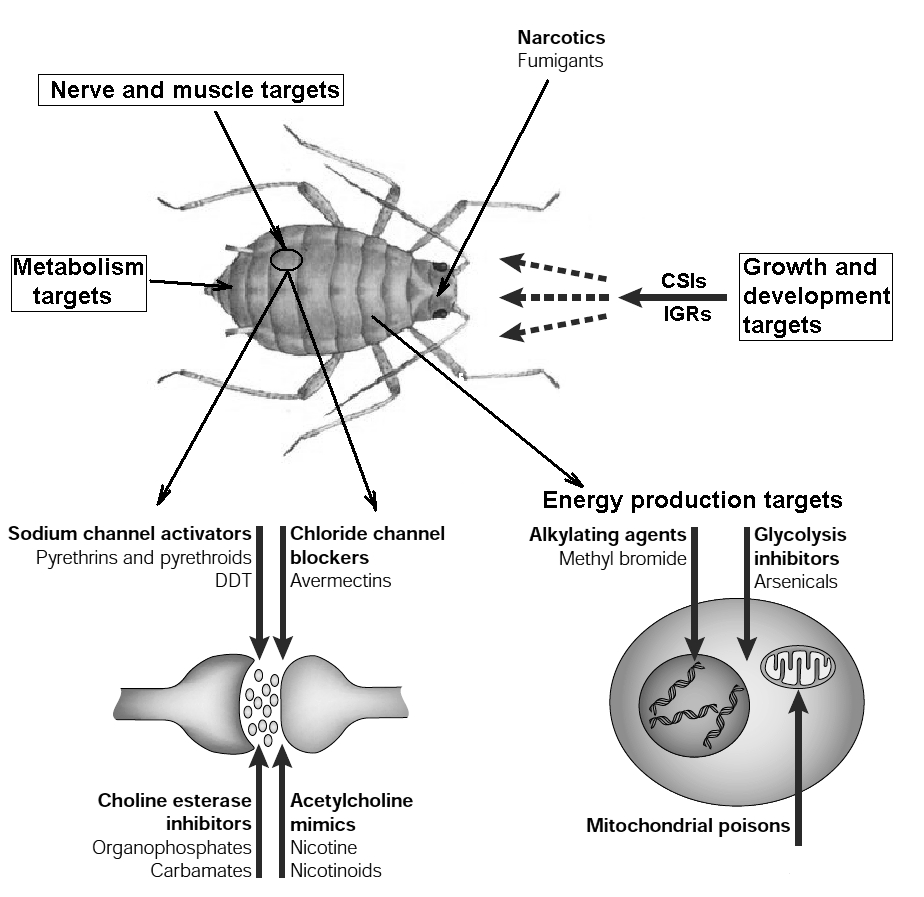
Insecticide sites of action. Some general methods of insecticide action are shown. Insecticides have many sites of action but most of those in common use affect the nervous system of the insect (*B.t.*, *Bacillus thuringiensis*; DDT, dichlorodiphenyltrichloroethane; CSIs, chitin synthesis inhibitors; IGRs, interference growth regulators) (Modified from Schneider (2000)).

*Insecticides* are any toxic substances used to kill insects. They are used primarily to control pests that infect cultivated plants or to eliminate disease-carrying insects. Based on their mechanisms of action, insecticides can be grouped in few principal ways (Figure 6(Fig. 6), Reference in Figure 6: Schneider, 2000[150]) (Jayaraj et al., 2016[84]; Liu et al., 2007[104]; Sparks and Nauen, 2015[162]): 

### Nerve and muscle targets

Cholinesterase inhibition. Carbamate and organophosphate insecticides are used to control insects via inhibition of cholinesterase leading to overstimulation of insect nervous system. Such inhibition of acetylcholine esterase finally kills animals. 

Acetylcholine receptor stimulation. Neonicotinoid insecticides and spinosad mimic the action of the neurotransmitter acetylcholine. They do not affect cholinesterase, but rather bind to acetylcholine receptors resulting in prolonged stimulation leading to insect death. 

Chloride channel regulation. There are three mechanisms: activation of chloride channels (avermectins), inhibition of gamma-aminobutyric acid (GABA) receptor (organochlorine insecticides), and agonists of the GABA-gated chloride channel (bifenazate).

Sodium channel modulators. Pyrethrins and pyrethroids bind to sodium channels fixing them in open state which leads to tremor, and eventually, to death.

### Growth and development targets

Chitin synthesis inhibitors. There are hormonal substances that inhibit the synthesis of chitin in insects and therefore result in death at early life stages during embryonic development or molting.

Insect growth regulators. Insecticides of this group disrupt endocrine system affecting in this manner production of hormones needed for animal growth and development into imago. Insects poisoned by insect growth regulators do not receive the signal to metamorphose. Some of them were designed to mimic effects of juvenile hormone necessary to enter metamorphosis. 

Nonspecific growth regulators. The exact mode of action of the growth regulators is not well understood. For example, hexythiazox kills before mite eggs can hatch and also kills some immature mites, but does not kill adult forms. 

### Energy production targets

Electron transport inhibition. Aliphatic type of organochlorine insecticides interferes with electron transport. They corrupt the ability of target organism to supply energy.

Oxidative phosphorylation disruption. Organotin miticides directly inhibit mitochondrial electron transport chain, whereas pyrroles uncouple electron transport and oxidative phosphorylation. This results in disability to produce ATP.

## Common Indicators of Pesticide Toxicity

It is impossible to monitor all anthropogenic influences that form potential threats to the environment. Therefore, the most promising approach to assess the overall quality of the environment is to examine biochemical responses that reflect the potential of contaminants impairing physiological processes in the exposed organisms (McCarthy and Shugart, 1990[119]). When a pesticide enters a living organism, it may be involved in metabolic processes due to which its toxic effects may be modulated. From this point of view, a search for potential appropriate biomarkers for pesticide toxicity should include common indicators of overall health as well as specific indices selected according to the mode of action of the investigated pesticide. It should be noted that selection of a reliable indicator for pesticide toxicity can be complicated if the mode of action of a toxicant is not known, or the presence of some other factors obscure the investigation (Niimi, 1990[131]).

Generally, it is believed that activities of biotransformation enzymes, which may be either induced or inhibited upon exposure to pesticides and other xenobiotics, are among the most sensitive intoxication biomarkers (Bucheli and Fent, 1995[19]). Many environmental contaminants and/or their metabolites have been shown to exert toxic effects such as inducing oxidative stress (Lushchak, 2011[107][109]; Winston and Di Giulio, 1991[191]). Reactive oxygen species are well-known side-products of certain metabolic pathways or the autoxidation of certain compounds and their concentrations may be acutely or chronically elevated under various conditions and cause the development of oxidative stress (Lushchak, 2011[107], 2014[111]). The cytotoxic effects of ROS are of particular interest since they may react with crucial cellular macromolecules, usually leading to effects including enzyme inactivation, lipid peroxidation, and DNA damage that, ultimately, can lead to cell death via necrosis or apoptosis (Winston and Di Giulio, 1991[191]).

Several hematological parameters, such as hematocrit or hemoglobin, protein or glucose concentration, although mainly nonspecific, may also be sufficiently sensitive indicators of certain types of pollutants to be considered as potential biomarkers for pesticide toxicity (Husak et al., 2014[79]; Husak, 2015[78]; Maksymiv et al., 2015[114]; Nieves-Puigdoller et al., 2007[130]).

Furthermore, when a pesticide possesses some genotoxic effect, it may induce a cascade of events such as formation of structural alterations in DNA, DNA damage and subsequent expression of mutant gene products, and diseases (e.g. cancer) resulting from damage to DNA, which also can be monitored for delineation of toxicity mechanisms (Shugart et al., 1992[157]). Detection and quantification of various events in this sequence also may be employed as biomarkers in organisms environmentally exposed to genotoxic substances (van der Oost et al., 2003[180]).

### Hematological and immunological parameters

Blood is a special organ which is quickly exposed to absorbed chemicals. Blood parameters are known to be highly informative indicators of organism status and have many advantages over other tissue samples (Ewald, 1995[50]). Samples of blood can be obtained regularly from test organisms, thus allowing the use of a non-destructive (vital) approach for effective assessment. In most cases, blood serves as a medium for signaling in animals. Typically, hematological parameters are non-specific in their responses towards chemical stressors (van der Oost et al., 2003[180]). Nevertheless, they may provide important information in effect-assessment studies. Disturbances in integrated functions can be detected, or strongly indicated, with rather simple analysis of blood parameters (Ewald, 1995[50]).

Blood indices can be divided into primary and secondary parameters. The primary blood components include formed elements (e.g. red and white blood cells) and plasma with diverse constituents. The latter include nutrients, ions, enzymes, and hormones (Niimi, 1990[131]). Hemoglobin content and hematocrit are common hematological indices that change in many model systems exposed to xenobiotics. Although their levels can also be influenced by biological factors like animal size, gender, and environmental factors like temperature and seasonality (Husak et al., 2014[79]; Kennedy, 1995[90]; Schlenk, 2005[149]), they are rather informative when dealing with pesticide intoxication (Kubrak et al., 2013[94]). Under stress conditions (including pesticide-induced) these parameters can be elevated to increase oxygen carrying capacity and the supply of oxygen to the major organs in response to higher metabolic demands (Rutten et al., 1992[146]). However, most investigators reported a decrease in hemoglobin and hematocrit in pesticide-treated animals indicating anemia, hemolysis and erythropoiesis dysfunction (El-Sayed et al., 2007[49]; Kubrak et al., 2013[94]; Saravanan et al., 2011[147]; Svobodová et al., 2003[170]; Vani et al., 2011[181]). Hemolysis in human erythrocytes caused by chlorophenoxy herbicides was reported by Duchnowicz et al. (2002[44]) as a result of free radical production by phenols (probably, due to autoxidation) or/and their direct attack on cell structure. In 2005 Duchnowicz and colleagues (2005[45]) found some protein damage in erythrocyte membranes which might result from the direct interaction of the investigated herbicide or an indirect effect, for example, via ROS-mediated oxidation.

Several immunological parameters may also be potentially used as biomarkers of stress conditions, e.g. white blood cell (leukocyte) and lymphocyte status (measured as a blood cell or differential counts), non-specific defense factors (such as lysosomal activity and levels of acute phase proteins in body fluids), etc. Differential changes in leukocyte counts may be a sensitive indicator of environmental stress. For example, differential changes in leukocyte counts were found to be reliable markers of the stress caused by environmental factors (Cole et al., 2001[30]). Decreases in lymphocyte numbers (lymphopenia) as a consequence of pesticide exposure have been reported for several fish species (Li et al., 2011[101]; Pimpão et al., 2007[138]; Svoboda et al., 2001[169]). Lymphopenia is often accompanied by concurrent increases in monocytes and neutrophils occurring in response to stress exposure (Kubrak et al., 2012[93]; Murad and Houston, 1988[126]).

Generally, immunological indices in the blood can supplement hematological parameters and help to clarify possible mechanisms of toxic impacts (Kubrak et al., 2012[93]; Li et al., 2011[101]). 

### Histological examinations

Histological changes are associated with complex biochemical and physiological responses to any stressor. Despite histopathological parameters are rather nonspecific and do not provide quantitative information, they are popular biomarkers for environmental pollution (Hinton and Lauren, 1990[72]). Being one of the most promising areas for assessing animal health and response to different chemical species, histological investigations include a wide range of studies that have generally indicated cellular differences between control and pesticide-exposed animals (Niimi, 1990[131]). It is generally assumed that histopathological biomarkers are valuable indicators of overall health status and that they reflect the total levels of pollution (van der Oost et al., 2003[180]). 

**Figure 7:**
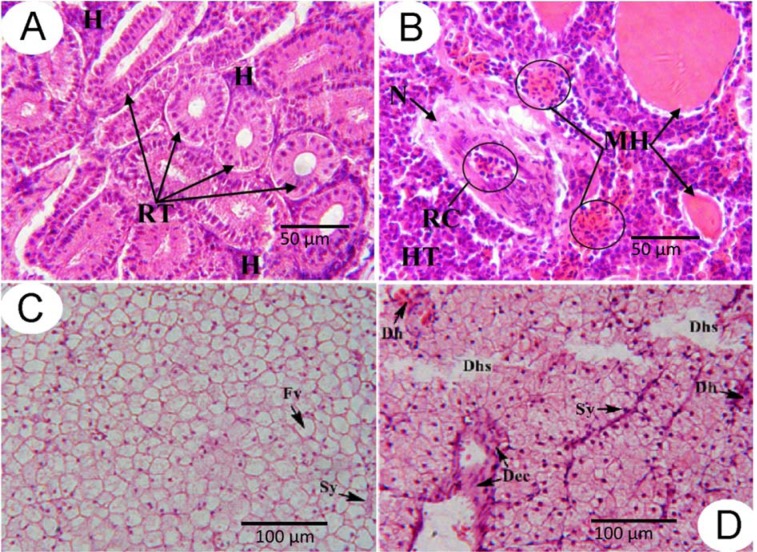
Histopathological alterations of the kidney (A and B) and liver (C and D) tissues of goldfish (*Carassius auratus* L.) is presented for exposure to control conditions (A and C) and 71.5 mg L-1 of Sencor for 96 h (B and D). Samples were stained with hematoxylin-eosin and photomicrographs were taken using 400× (for kidney) and 200× (for liver) magnification. RT - renal tubules; H - hematopoietic tissue; N - necrotic cells and nuclei of tubular epithelium; MH - multiple hemorrhage; HT - hypertrophy of intertubular hematopoietic tissue; RC - red blood cells in necrotic tubules and Bowman's capsule; Fv - fatty vacuolization; Sy - sinusoids; Dh - detachment of endothelial cytoplasm with diffuse hemorrhage; Dhs - dystrophy of hepatic cells; Dec - detachment of endothelial cytoplasm (modified from Husak et al. (2014), Maksymiv et al. (2015)).

However, histological changes are not as easily and objectively assessed as are biochemical markers and may require substantial experience by the researcher (Ewald, 1995[50]). The results of our studies, in particular the histopathological changes, indicate that goldfish exposure to the triazine herbicides, Sencor and Gesagard, over 96 h caused severe deleterious effects which could be related to liver and kidney dysfunction (Hodgson and Goldstein, 2001[74]; Maksymiv et al., 2015[114]; Mosiichuk et al., 2015[123]). The histological analysis of control fish showed normal liver and kidney structures (Figure 7A and 7C(Fig. 7); References in Figure 7: Husak et al., 2014[79]; Maksymiv et al., 2015[114]). However, exposure for 96 h to 71.5 mg L^−1^ of Sencor increased the number of dilated sinusoids (Figure 7D(Fig. 7)). Dystrophy in hepatic cells and detachment of endothelial cytoplasm was observed along with an increased number of dilated sinusoids at this Sencor concentration. The treatment induced various histopathological changes in goldfish kidney, such as hypertrophy of intertubular hematopoietic tissue, small and multiple hemorrhages, glomerular shrinkage, a decrease in space between glomerulus and Bowman's capsule, degeneration, and necrosis of the tubular epithelium (Figure 7B(Fig. 7)). 

Also, results from histological examinations do not show a direct influence of the pollutant, so they should be considered together with other parameters. Moreover, they do not provide reliable quantitative parameters and researchers have to use them as semi-quantitative data.

### Biochemical indices

In some cases, xenobiotic biotransformation can result in the formation of compounds that may be more easily monitored than the original chemical and thus such products may be used as a biomarker of pesticide exposure. Biomonitoring using biotransformation products of xenobiotics requires knowledge of the extent of metabolic conversion and the types of metabolic products formed for each particular compound/s produced by the organism (van der Oost et al., 2003[180]). However, frequently we do not know the nature of the compounds formed. In this case, a search for potential biochemical markers of pesticide exposure should be focused on measurements of key metabolic parameters of a particular pathway, because many changes induced by pesticide exposure lead to metabolic disturbances, inhibition of important enzymes, growth retardation, etc. (Murty, 1986[127]). From this point of view, main metabolic parameters, such as glucose concentration or activities of serum enzymes, become of great interest. In some cases, somatic (e.g. growth rate) and behavioral measures can also be effective. The tests often require much work and take time to perform, but can provide very valuable information (Ewald, 1995[50]).

It has been suggested that, in general, stress induces elevation of the transamination pathway (El-Sayed et al., 2007[49]) and the activities of plasma alanine aminotransferase (ALT) and aspartate aminotransferase (AST) have been used as relevant stress indicators (Ishikawa et al., 2007[81]). Increases in ALT or AST activities in the extracellular fluid or plasma are sensitive indicators to minor cellular damage since the levels of these enzymes within healthy cells always substantially exceed those in the extracellular fluids (Moss et al., 1986[124]). Monosex tilapia acutely exposed to a deltamethrin-based pesticide demonstrated significantly increased activities of these serum transaminases (El-Sayed et al., 2007[49]). Also increased plasma ALT activity was found in our previous investigation in goldfish exposed to 2,4-D herbicide (Kubrak et al., 2013[94]) and interpreted as a possible evidence of hepatotoxicity and damage to other tissues investigated.

Lactate dehydrogenase (LDH) is a glycolytic enzyme recognized as a potential biomarker for assessing chemical toxicity (Kubrak et al., 2013[94]; Li et al., 2011[101]). Elevated plasma LDH was reported by Li et al. (2011[101]) in response to verapamil exposure of juvenile rainbow trout (*O. mykiss) *and might be explained by the release of LDH from injuried tissues (Mishra and Shukla, 2003[121]). A significant increase in LDH activity in the serum of *Cyprinus carpio* exposed to 2,4-diamin was also reported by Oruç and Ūner (1999[134]) along with increased serum glucose and liver glycogen levels and decreased glycogenolysis and glycolysis. Overall, this indicated significant effects of 2,4-diamin on carbohydrate metabolism.

As described earlier, it is generally accepted that there are three phases in xenobiotic detoxification. In phase I many xenobiotics are enzymatically modified to hydrolyze or introduce reactive and polar groups (such as hydroxyl) onto the molecule. In majority cases, phase I involves transformation of xenobiotic compounds by microsomal monooxygenase enzymes, also known as the mixed-function oxidase (MFO) system (i.e. cytochrome P450). The system facilitates the excretion of certain compounds by transforming lipophilic xenobiotics to more water-soluble compounds (Bucheli and Fent, 1995[19]). Since the mixed-function oxidase system is sensitive to certain environmental pollutants, its activity may serve as a marker for exposure to certain classes of pesticides (Bucheli and Fent, 1995[19]).

Phase II detoxification enzymes catalyze conjugation of xenobiotics (usually after hydroxylation) with several endogenous compounds (e.g. GSH, sulfate, glycine, or glucuronic acid), thus facilitating excretion of the chemicals by the addition of more polar groups to their structures (Commandeur et al., 1995[31]). Phase II enzymes can play an important role in homeostasis as well as in detoxification and clearance of many xenobiotics (van der Oost et al., 2003[180]). Conjugation with GSH is the major pathway for processing of electrophilic compounds and their metabolites (George, 1994[60]), due to which GSH levels can be used as another potential biomarker for pesticide toxicity. As an important antioxidant, GSH is involved in the enzymatic and non-enzymatic protection against ROS and detoxification of endogenous and exogenous toxicants (i.e. pesticides) via reaction with electrophilic compounds to replace hydrogen, chlorine, or nitro-groups (Lushchak, 2012[112]; Stegeman et al., 1992[165]). In this case, changes in the levels of different glutathione forms (either reduced GSH or oxidized GSSG) may indicate a shift in the prooxidant-antioxidant balance, which often takes place under pesticide-induced stress conditions (Atamaniuk et al., 2013[3]; Lushchak, 2012[112]; Maher, 2005[113]). Hence, an increase in glutathione levels is likely to provide increased protection of cells from both ongoing stress and subsequent, more severe stress (Maher, 2005[113]), whereas elevation of oxidized glutathione (GSSG) or the ratio [GSSG]/[total GSH] is used as an evidence of oxidative stress (Lushchak, 2012[112]; Zhang et al., 2004[192]). A decrease in glutathione thiol status, i.e. the ratio of reduced to oxidized glutathione, due to either direct ROS scavenging or increased glutathione peroxidase/transferase activity is perhaps the most obvious direct effect of certain pollutants (Otto and Moon, 1995[135]; Stegeman et al., 1992[165]). Alternatively, a normal ratio [GSSG]/[total GSH] can be maintained due to increased activities of glutathione reductase or increased glutathione synthesis (van der Oost et al., 2003[180]). Conjugation of electrophilic compounds with GSH is substantially accelerated by GSTs (Lushchak, 2012[112]) and the toxicity of many exogenous compounds can be modulated by induction of GSTs (van der Oost et al., 2003[180]).

Animal treatment with pesticides is often accompanied by the development of oxidative stress (Atamaniuk et al., 2013[3]; Kubrak et al., 2013[94]; Lushchak, 2011[107][109]; Matviishyn et al., 2014[118]). Antioxidant enzymes play key roles in ROS detoxification. Therefore their activities are believed to be good markers of perturbations in ROS homeostasis frequently affected by environmental toxicants (van der Oost et al., 2003[180]; Lushchak, 2016[108]). At the same time, these enzymes are sensitive to many factors and show diverse responses to various environmental and chemical stresses (Hermes-Lima, 2004[71]; Lushchak, 2011[107][109]; Storey, 1996[167]; Tseng et al., 2011[175]).

Zhang and colleagues (2004[192], 2005[193]) described the effects of 2,4-dichlorophenol (2,4-DCP) on antioxidant indices in goldfish liver after a 40-day exposure. The authors found a significant increase of superoxide dismutase activity at low/intermediate concentrations of 2,4-DCP perhaps due to early adaptation suggesting that this parameter could be a potential biomarker of fish exposure to 2,4-DCP (Zhang et al., 2004[192], 2005[193]). Goldfish exposure to mancozeb-containing carbamate fungicide Tattoo for 96 h also enhanced liver and renal SOD activity (Atamaniuk et al., 2013[3]). Catalase activity is also an important indicator of pesticide-induced oxidative stress (Manda et al., 2009[115]; Shi et al., 2005[155]) particularly aminotriazole inhibited catalase in goldfish tissues (Lushchak, 2011[109]; Vasylkiv et al., 2011[182]), whereas the activity was enhanced in liver of Tattoo-treated goldfish (Atamaniuk et al., 2013[3]). The activity of Se-dependent glutathione peroxidase (Se-GPx) was also increased under fish exposure to 2,4-DCP and was proposed as a potential biomarker (Zhang et al., 2005[193]).

Additionally, if pesticides are involved in oxidative stress development, they directly or indirectly increase ROS steady-state level (Atamaniuk et al., 2013[3]; Kubrak et al., 2013[94]). Therefore, commonly used indices of ROS damage to biomolecules may be important. Protein carbonyl groups are frequently quantified among such indices and indicate ROS-induced protein oxidation in tissues (Dean et al., 1997[37]; Lushchak, 2007[110]). Enhanced levels of protein carbonyls indicate a potentially increased ROS steady-state concentration under pesticide influence (Atamaniuk et al., 2013[3]; Li et al., 2010[103], 2011[101]). Measurements of levels of lipid peroxides may be used similarly (Calabrese et al., 2000[23]; Lushchak, 2012[112]). 

With respect to neuromuscular functions, recent studies indicated that the “old” classic biomarker, AChE, that is sensitive to organophosphate (OP) and carbamate pesticides, may respond to low levels of contaminants in the environment and be successfully used in the toxicity monitoring (Liu et al., 2007[104]; Valbonesi et al., 2011[179]; Vani et al., 2011[181]). Inhibition of AChE results in a buildup of acetylcholine levels, causing a continuous and excessive stimulation of the nerves and muscle fibers, which leads to tetany, paralysis, and eventual death (Liu et al., 2007[104]). Measurement of AChE inhibition is one of the most widely used biomarkers of environmental pollution with pesticides (Atamaniuk et al., 2013[3]; Edwards et al., 1991[47]; Matviishyn et al., 2014[118]; Vani et al., 2011[181]).

All of the biochemical indices mentioned above are very important in the investigation of hazardous influences of pesticides, but the relevant list of biomarkers may become wider or narrower in each particular study depending on the mode of action and metabolic processes of the chemicals under inspection. Therefore, these and many other biochemical parameters help to clarify the possible mechanisms of the toxic impacts of pesticides.

### Genetic studies

Direct or indirect interaction of pesticides with DNA leads to damage of the latter or chromosomal aberrations are also effective indicators of pesticide toxicity within the context of carcinogenesis and teratogenesis (Calviello et al., 2006[24]; González et al., 2005[65]). They are studied in the field of genetic toxicology and can be analyzed by distinctive kinds of genotoxicity tests. Genetic toxicology can be defined as the study of pollutant-induced changes to the genetic material of organisms and a growing body of data concerning the genetic toxicity of pesticides has been collected from epidemiological and experimental studies that examine parameters including chromosomal aberrations, formation of micronuclei, cell-cycle progression, sister chromatid exchanges and DNA strand breaks (comet assay) (Bolognesi, 2003[14]). For example, a recent study of the pesticide genotoxic effects of an atrazine-based herbicide on a model fish, *Carassius auratus* L., showed a significant increase in the frequencies of micronuclei and DNA strand breaks in goldfish erythrocytes, indicating the genotoxic potential of this pesticide (Cavas, 2011[26]). DNA damage under 2,4-D exposure of CHO (Chinese hamster ovary) cells also provided additional evidence for the genotoxicity of pesticides (González et al., 2005[65]). All methods applied to demonstrate high efficiency and may be considered as potential biomarkers of pesticide genotoxicity (Bolognesi, 2003[14]; González et al., 2005[65]; van der Oost et al., 2003[180]).

## Principal Molecular Mechanisms of Pesticide Toxicity

The problem of the toxicity of pesticides and other related chemicals to non-target organisms is still a major concern around the world. Since pesticides may produce many physiological and biochemical changes when they enter the body, a search for mechanisms of their toxicity can be much more complicated than expected. Perhaps the pesticide mode of action may be one of the most reliable tools for searching the mechanisms of their toxicity.

Pesticides can cause adverse effects by interfering with the body's hormones or messengers (Khan and Law, 2005[91]), affecting the nervous system (e.g. organochlorine pesticides) (Bolognesi and Merlo, 2011[15]), or directly or indirectly inducing changes in the activities of certain enzymes (Atamaniuk et al., 2013[3]; Kubrak et al., 2012[93], 2013[94]; Matviishyn et al., 2014[118]). A large group of pesticides may directly enhance ROS levels in the living organisms due to their autoxidation by molecular oxygen (Bolognesi and Merlo, 2011[15]; Lushchak, 2011[109]). Mostafalou and Abdollahi (2013[125]) have conducted extensive work to systematically catalog the molecular mechanisms of pesticide toxicity. Their study resulted in a theoretical interpretation of causal relationships between pesticide exposure and human chronic diseases via DNA damage (Mostafalou and Abdollahi, 2013[125]).

### Molecular mechanisms of pesticide-induced neurotoxicity 

The nervous system is the main target of acute toxic action by diverse organochlorine insecticides. These chemicals are the active ingredients of various home and garden products as well as some agricultural and environmental pest control products; their high environmental persistence makes them dangerous contaminants (Bolognesi and Merlo, 2011[15]; Rizzati et al., 2016[144]). Some of them, such as derivatives of the banned pesticide DDT have been shown to induce neural cell death by apoptosis through the activation of mitogen-activated protein kinases (Shinomiya and Shinomiya, 2003[156]). Toxicity of DDT and pyrethroids was found to be associated with blocking of voltage-gated sodium channels (VGSCs) in plasmatic membrane of neurons (Silver et al., 2017[159]). The neurotoxic effect of endosulfan is probably realized via its well-known ability to block neuronal GABAA-gated chloride channels (Kamijima1 and Casida, 2000[87]).

**Figure 8:**
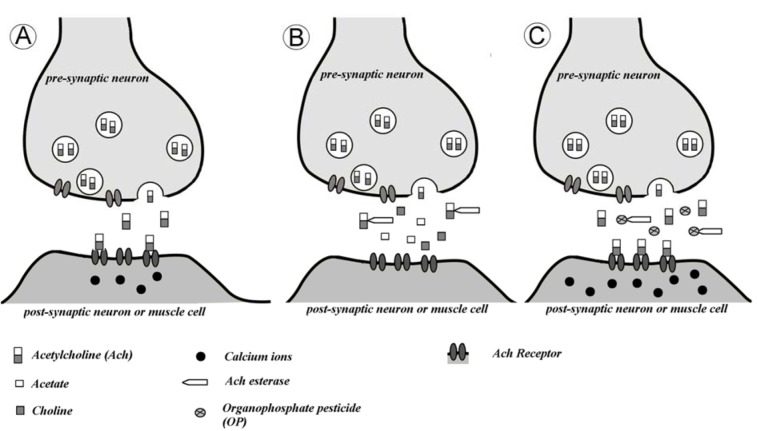
Effect of organophosphorus insecticides in the transmission of nerve impulses: A - acetylcholine signaling at synapse; B - acetylcholinesterase stops signaling process; C - organophosphates inhibit acetylcholinesterase.

Organophosphorus pesticides are also potent neurotoxins since they are irreversible inhibitors of acetylcholinesterase (Figure 8(Fig. 8)) (Galloway and Handy, 2003[57]). Most animals also possess non-specific esterases or pseudocholinesterases with high affinity for butyrylcholine. Fish brain, for example, contains AChE, but not BChE, whereas muscle tissues contain both AChE and BChE (Sturm et al., 2000[168]). AChE is involved in the deactivation of acetylcholine (hydrolysis to choline and acetate) at nerve endings, preventing continuous nerve firing, and is vital for normal functioning of sensory and neuromuscular systems (van der Oost et al., 2003[180]). The activity of AChE has been widely used in aquatic animals to diagnose exposure to organophosphate or carbamate pesticides (Fulton and Key, 2001[56]). In the synapse, acetylcholinesterase catalyzes degradation of acetylcholine (Figure 8B(Fig. 8)). Organophosphate pesticides phosphorylate acetylcholinesterase decreasing its activity (Figure 8C(Fig. 8)). In result, acetylcholine accumulates in the central and peripheral nervous systems. Such inhibition provokes an accumulation of acetylthiocholine in synapses with disruption of the nerve function that can end in the death of the organism (Peakall, 1992[136]). 

Chlorpyrifos, an organophosphate insecticide, is known to inhibit AChE by phosphorylating the enzyme in both neuron synapse and plasma and it can cause symptoms such as nausea, dizziness, and confusion, and even hyperactivity, paralysis, respiratory paralysis, and death at exposure to pesticide at high concentrations (John and Shaike, 2015[86]). Not like most organochlorine pesticides, it is relatively nonpersistent and its principal degradation products are less toxic than the parent chemical. Interestingly, aquatic and terrestrial microorganisms and plants are rather tolerant to chlorpyrifos, whereas aquatic invertebrates, particularly crustaceans and insect larvae, are very sensitive to exposure (Barron and Woodburn, 1995[11]). Diazinon, another organophosphate, also inhibits AChE (Bisson and Hontela, 2002[13]).

A lot of studies verifying the neurotoxicity of 2,4-dichlorophenoxyacetic acid (2,4-D) have been focused on the central nervous system (Rosso et al., 2000[145]). This toxicity is caused, in part, by the formation of free radicals and leads to decreased GSH levels and impaired action of antioxidant enzymes such as superoxide dismutase and catalase (Bukowska, 2003[20]). Bernard et al. (1985[12]) found decreased AChE specific activity after 2,4-D injection in some rat muscles *in vivo*, but not *in vitro* indicating a nondirect effect unlike that of OPs. Others found that 2,4-D might also affect the enteric nervous system by documenting atrophy of the cellular body in a general population of rat myenteric neurons and triggered by 2,4-D hypertrophy of the cell body of neurons positive to NADPH-diaphorase (Correa et al., 2011[34]).

Many dithiocarbamates are implicated in inducing peripheral Parkinson's-like neuropathy (Rath et al., 2011[141]). These chemicals induce intraneuronal oxidative stress leading to neuronal damage, since metal ions released during cell damage may promote lipid peroxidation and enzyme inhibition resulting in neurotoxic effects (Fitsanakis et al., 2002[53]; Nobel et al., 1995[132]). The fungicide, maneb, affects biological systems in numerous ways, but its primary neurotoxic mechanism is still under debate (Meco et al., 1994[120]). The compound impairs the operation of some receptors and ion channels of the plasma membrane, systems for signal transduction and second messenger synthesis, and some cellular enzymes and metalloproteins. The mechanisms that have been suggested to explain the maneb neurotoxicity include dopamine autoxidation, stimulation of ROS generation, a decrease in the levels of GSH and reduced activities of glutathione peroxidase and catalase (Meco et al., 1994[120]). Degeneration of nigrostriatal dopaminergic neurons is often associated with a late onset of the progressive neurological disorder - Parkinson's disease, the occurrence of may be linked with influence of pesticides as an environmental factor (Bolognesi and Merlo, 2011[15]). Indeed, Parkinsonian symptoms occurred following exposure to the herbicide paraquat or the fungicide maneb. Paraquat is thought to be transported across the blood-brain barrier by the action of a neutral amino acid transporter such as the system L carrier (LAT-1), which normally carries L-valine and L-phenylalanine; indeed, administration of high levels of these amino acids has been reported to prevent paraquat-induced neurotoxicity (Chanyachukul et al., 2004[27]). The mitochondrial complex I inhibitor, rotenone, enhances mitochondrial ROS production resulting in dopamine redistribution to the cytosol and may potentiate rotenone-induced apoptosis of dopaminergic cells (Watabe and Nakaki, 2007[186]).

### ROS-mediated pesticide toxicity

Many pollutants including pesticides may exert toxicity related to induction of oxidative stress (Lushchak, 2011[107][109], 2016[106]; van der Oost et al., 2003[180]; Wang et al., 2016[185]). This induction can take place in several ways:

1. Certain chemicals may increase ROS production as byproducts of the operation of detoxification pathways;
2. Some pesticides can alter the operation of the mitochondrial and endoplasmic reticulum electron transport chains leading to ROS overproduction;
3. Pesticides can also increase ROS production by entering redox cycles (e.g. autoxidation), which has been proposed as the central mechanism for the toxic effects of many environmental toxicants including pesticides;
4. Pesticide can also inhibit antioxidant and associated enzymes or the biosynthesis of antioxidants such as glutathione.

The ability to act as a prooxidant agent is one of the possible mechanisms to explain the toxicity of DTC fungicides and several reports have recently provided support to this hypothesis. In particular, maneb and zineb were shown to catalyze cathecol autoxidation (Fitsanakis et al., 2002[53]), and the mancozeb-containing pesticide Tattoo induced oxidative stress in goldfish and frogs, altering activities of primary antioxidant enzymes (Atamaniuk et al., 2013[3]; Falfushinska et al., 2008[52]). The presence of metals in the chemical structure of these pesticides can catalyze ROS formation via the Fenton reaction that could explain the observed prooxidant activity (Calviello et al., 2006[24]; Lushchak, 2016[106]). Interestingly, oxidative stress along with toxic effects was noted in various species of animals, such as rats, mice, zebrafish, clams, and sea snail, Hexaplex* trunculus*, suggesting that oxidative stress induced by permethrin might be a common mechanism for its toxicology (Wang et al., 2016[185]). Maneb can also induce oxidative stress as evidenced by the formation of additional carbonyl groups in proteins and α-synuclein aggregation due to proteasomal dysfunction, which was shown to be modulated by intracellular glutathione (Barlow et al., 2005[9]). Mancozeb, thiram, and disulfiram also caused membrane potential changes, impaired ATP-dependent glutamate uptake into the synaptic vesicles, and prevented binding of glutamate to its receptors resulting in excitotoxic effects in the brain (Vaccari et al., 1999[178]). Generally, the prooxidant properties of DTC compounds cause imbalances between GSH and GSSG, typically raising levels of GSSG (Burkitt et al., 1998[22]). Increased levels of GSSG can lead to the activation of the transcription factor nuclear factor kappa B (NF-κB) that in turn stimulates a stress and inflammatory response and affects cell survival (Delhalle et al., 2004[38]). On the other hand, reduction of GSSG to GSH catalyzed by glutathione reductase was inhibited by DTC which was found to inactivate several different transcription factors principally, the NF-κB and hypoxia-inducible factor HIF-1α (Haddad, 2003[69]). A number of enzymes including heme oxygenase, cytochrome P450, superoxide dismutase, glutathione reductase, caspase, etc. are inhibited by DTC (Dalvi et al., 2002[36]; Seefeldt et al., 2009[152]). On the other hand, DTC pesticides are also capable of acting as antioxidants. They react with hydroxyl radicals, peroxides, and superoxide ions to decrease their oxidative activity (Liu et al., 1996[105]; Nobel et al., 1995[132]). Additionally, the DTC compounds can form mixed disulfides with other molecules containing thiol functional groups such as proteins, peptides and enzymes modulating their biological activities. Covalent modification of cysteine residues in the active sites can affect enzyme activities (Lushchak, 2012[112], 2016[108]; Rath et al., 2011[141]).

The activities of antioxidant enzymes, in turn, can be affected by pesticides. Superoxide dismutase (SOD) plays a pivotal antioxidant role as evidenced by its presence in virtually all aerobic organisms examined to date (Lushchak, 2011[107][109]; Stegeman et al., 1992[165]). Catalases are known to facilitate the removal of hydrogen peroxide, which is metabolized by them to molecular oxygen and water. Glutathione peroxidase (GPx), which employs GSH as a cofactor, reduces many organic peroxides; its operation plays an especially important role in protecting the integrity of membranes under oxidative insults via prevention of lipid peroxidation (Stegeman et al., 1992[165]; van der Oost et al., 2003[180]). Lipid peroxidation or oxidation of polyunsaturated fatty acids is a very important consequence of ROS attack to living organisms since it demonstrates the ability of a single radical species to propagate a number of deleterious biochemical reactions (Stegeman et al., 1992[165]; van der Oost et al., 2003[180]).

It is known that some pesticides can cause oxidative stress by stimulating ROS generation (Banerjee et al., 1999[7]). Therefore, they are suspected to induce alterations in antioxidant and ROS-scavenging enzymatic systems. Pesticide-induced toxicity of many pollutants may be realized via stimulation of peroxidation of lipids (Akhgari et al., 2003[2]). For example, long-term exposure to propiconazole was shown to cause ROS-promoted stress in several tissues of rainbow trout, *O. mykiss*, reflected by significantly higher levels of lipid peroxides and protein carbonyl groups (Li et al., 2010[103]). 

Production of ROS can also be stimulated by phenoxy herbicides perhaps due to ROS formation by autoxidation, or a direct attack of the phenoxyl radicals on sensitive enzymes from a number of metabolic pathways (Selassie et al., 1998[153]). Indeed, several studies demonstrated that 2,4-D induced oxidative stress and depleted antioxidants both *in vitro* and *in vivo* (Bukowska, 2003[20]; Kubrak et al., 2013[94]; Matviishyn et al., 2014[118]; Wafa et al., 2012[184]).

**Figure 9:**
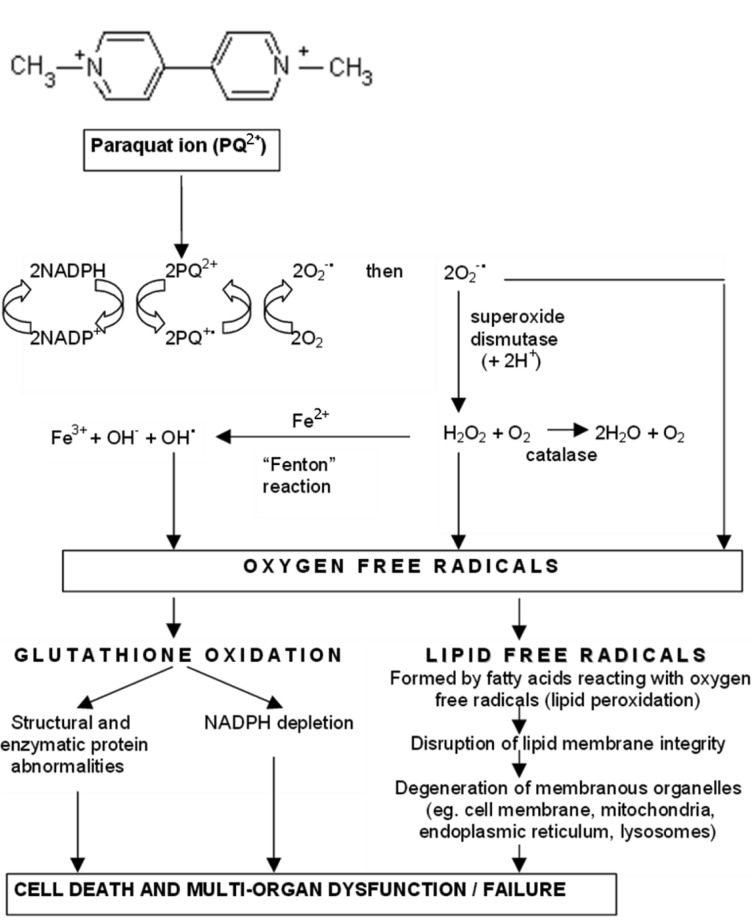
Paraquat-induced oxidative stress. Description in the text. (Modified from Dinis-Oliveira et al. (2008))

Herbicide paraquat can enter an autoxidation process resulting in the production of superoxide anion radicals (Figure 9(Fig. 9); Reference in Figure 9: Dinis-Oliveira et al., 2008[41]) (Bonneh-Barkay et al., 2005[17]). As a free radical generator, paraquat has a redox potential of -446 mV. Formed superoxide anion may be converted to hydrogen peroxide and hydroxyl radical (Figure 9(Fig. 9)). Pulmonary effects as the result of ROS generation with extensive oxidative damage represent the most lethal and least treatable manifestation of paraquat toxicity in exposed animals (Bolognesi and Merlo, 2011[15]). Despite its structural similarity to paraquat, the mechanism of diquat toxicity was found to differ. It has been proposed that diquat stimulates ROS production by inhibition of complexes I and III of the mitochondrial electron transport chain (Drechsel and Patel, 2009[42]).

In redox cycles, the parent compound is typically first reduced enzymatically by a NADPH-dependent reductase to yield a xenobiotic radical (van der Oost et al., 2003[180]). This radical donates its unshared electron to molecular oxygen, yielding an O_2_•^− ^radical and the parent compound. Thus, at each turn of the cycle, two potentially deleterious events occur: reductant oxidation and oxyradical formation (Goeptar et al., 1995[63]). These processes induce either adaptive responses, such as increase in the activities of antioxidant enzymes and concentrations of low molecular mass antioxidants like glutathione, or manifestations of oxidant-mediated toxicity such as oxidations of proteins, lipids and nucleic acids, as well as perturbing tissue redox status (Lushchak, 2011[107][109], 2016[108]).

### Endocrine and reproductive disruptions under pesticide influence

A number of pesticides, such as vinclozolin, dicofol, atrazine, and others, belong to the class of chemicals called endocrine disruptors (EDCs) that are known to interfere with the production, release, transport, metabolism, action, or elimination of hormones responsible for maintenance of homeostasis and regulation of developmental processes (Bolognesi and Merlo, 2011[15]; Khan and Law, 2005[91]). In fish, EDCs can cause male fish to transform into ones with female characteristics. These outward symptoms of developmental disruption are accompanied by reduced fertility or even sterility in adults, as well as lower hatching rates and viability of offsprings (Ewing, 1999[51]; Goodbred et al., 1997[66]). Pesticides as the exogenous hormone agonists/antagonists can disrupt the function of endogenous hormones. Agonists may interact with hormone-binding proteins, and antagonists may displace endogenous hormones (Tollefsen, 2002[173]). Some toxicants also disrupt the synthesis of hormone receptors (Scott and Sloman, 2004[151]). 

The ultimate aim of reproduction is birth and its success depends upon both male and female reproductive systems (Gupta, 2011[68]). The decreased reproductive capability may be considered as one of the most damaging effects of the persistent pollutants (e.g. pesticides) released by humans (van der Oost et al., 2003[180]). The presence of these chemicals in the environment has become a global concern. As blockers of sex hormone effects, pesticides may cause abnormal sexual development and other disturbances of other vital processes (Ewing, 1999[51]; Gupta, 2011[68]).

Although dioxin is not used as pesticide, in some cases it can arise as a contaminant at production of herbicides (Manz et al., 1991[117]). Dioxin is the most toxic and best-studied chemical that can lead to male reproductive toxicity (Gupta, 2011[68]). It causes functional developmental toxicity with additional structural abnormalities that are delayed in their appearance in multiple species. The effects of prenatal dioxin exposure on the reproductive system of female rats and hamsters indicated a delay in vaginal opening and clefting of the external genitalia (Cooper et al., 2000[33]). This may reflect a lack of appropriate differentiation.

The herbicide atrazine has been proposed to exert adverse effects on the reproductive system of animals including mammals, fish, and amphibians (Grasiela and Silva-Zacarin, 2012[67]). Indeed, it is reported to disrupt ovarian function by altering hypothalamic control of the pituitary and the release of luteinizing hormone and prolactin in female rats (Cooper et al., 2000[33]). Ovarian cycle irregularities may be due to the ability of atrazine to interfere with hormone synthesis, binding to the estrogen receptor without activation (Solomon et al., 2008[160]). Similar effects were observed when atrazine degradation products were found to affect the onset of puberty and thyroid function in male rats via actions on the central nervous system and its subsequent control of the pituitary-gonadal axis (Stoker et al., 2002[166]). There was also evidence of reproductive function impairment and depletion of the antioxidant defense system in rat testis and epididymis after exposure to atrazine (Grasiela and Silva-Zacarin, 2012[67]).

It was observed that a single dose of benzimidazole carbamates induced rapid testicular effects, detectable as an increase in testis weight, but having long-term effects leading to testicular atrophy and infertility. The inflammatory response with occluded ductules caused subsequent damage to the ductal epithelium. The rete testis was swollen with compacted sperm and the seminiferous tubules were atrophic with edematous interstitial space (Nakai and Hess, 1997[128]).

Dithiocarbamates are well-known endocrine disrupters that alter thyroid hormone levels and animal mass. The number of healthy follicles was a significantly decreased, whereas thyroid gland weight was increased (Baligar and Kaliwal, 2001[5]). The hypothyroid and antithyroid effects of the fungicides zineb and mancozeb are associated with their metabolite ethinylthiourea (Houeto et al., 1995[76]). The action of ethinylthiourea on the thyroid gland with resultant hyperplasia and a decrease in thyroid hormone levels is the most prominent aspect of its toxicity (Houeto et al., 1995[76]). 

Interestingly, ROS are not only associated with oxidative stress, but are also thought to play significant roles in reproduction (Bongiovanni et al., 2012[16]). Hence, induction of oxidative stress by pesticides has also been pointed out as a possible mechanism of some toxic effects on the reproductive system (Abdollahi et al., 2004[1]).

### Carcinogenic, teratogenic, and genotoxic effects of pesticide exposure

Animal studies remain a valuable tool for detecting of potential human cancer hazards. However, the evidence that a chemical causes tumors in experimental animals is considered sufficient only when experimental data show an increased incidence of malignant tumors in multiple species and following multiple routes (Bolognesi and Merlo, 2011[15]).

A few pesticides (e.g. dithiocarbamates) have been demonstrated to be animal carcinogens. The production of carcinogenic compounds such as N-nitrosocarbaryl, a derivative of carbaryl which is a potent carcinogen in rats is the principal hazard of these toxins (Bolognesi and Merlo, 2011[15]). Ethylene thiourea, a degradation product of ethylene bisdithiocarbamate fungicides, is also reported to be a teratogen, goitrogen, and carcinogen that can disrupt thyroid function and is causally related to thyroid cancer in animals (Steenland et al., 1997[164]). Thyroid-like effects of fungicides were observed in subchronic level studies of metiram-treated rats as evidenced by increased thyroid mass, increased levels of thyroid-stimulating hormone and decreased levels of T4 (serum thyroxin) (U.S. EPA, 2005[177]). The ethylene-bis-dithiocarbamates, in general, are considered to be carcinogenic because of their metabolite ETU (Houeto et al., 1995[76]; U.S.DA, 1998[176]). *In vitro* studies of zineb effects on human lymphocytes and CHO cells showed induction of DNA strand breaks suggesting its carcinogenic potential in the event that the affected cells survived and propagated (González et al., 2003[64]; Soloneski et al., 2002[161]). Calviello et al. (2006[24]) demonstrated DNA single-strand breaks in rat fibroblasts exposed to mancozeb.

Atrazine is one of the most important triazine herbicides used in large quantities worldwide. It has been identified as an endocrine disrupting chemical and a potential carcinogen (Chelme-Ayala et al., 2005[28]). The appearance of mammary tumors in atrazine-treated female rats has been documented (Eldridge et al., 1999[48]).

Genotoxic compounds are those that act through direct or indirect DNA damage (Bolognesi and Merlo, 2011[15]). Many pesticides tested induced diverse mutations via DNA damage. The genotoxic potential of agrochemical ingredients is generally low, but occupational exposure to mixtures of pesticides has been associated with an increase in genotoxic damage in a number of studies (Calviello et al., 2006[24]; González et al., 2005[65]).

In laboratory tests, chlorothalonil fungicide caused kidney damage, anemia, liver damage, embryo loss during pregnancy, oxidative DNA damage, and cancers of the kidney and forestomach (Oruc, 2010[133]). Most of these effects have been observed in several tested species. As a result, chlorothalonil is classified as a “probable human carcinogen” by the U.S. Environmental Protection Agency (Cox, 1997[35]). 

Pesticide-induced oxidative stress is well-known to cause genotoxicity (Franco et al., 2010[54]). In general, pesticides have been shown to alter cellular redox balance enhancing ROS levels, lipid peroxidation, and depletion of antioxidant defenses (Abdollahi et al., 2004[1]; Banerjee et al., 2001[8]; Lushchak, 2016[108]). Living organisms protect themselves from genotoxic and carcinogenic compounds in different ways. GSTs are an enzyme superfamily responsible for GSH conjugation with xenobiotics in their original or transformed forms and they might protect organisms against exogenous and endogenous toxicants (Lushchak, 2012[112]). The activity of these enzymes in many cases can be altered by pesticide exposure (Atamaniuk et al., 2013[3]; Kubrak et al., 2012[93]). 

It is known that the herbicide 2,3,7,8-tetrachlorodibenzodioxin is also an environmental teratogen. It affects cellular immunity in rodents and alters reproductive functions in the immature rat model through influences on the hypothalamic-pituitary axis as well by direct effects on the ovary (Li et al., 1995[100]). Teratology studies on 2,4-D indicated that malformations are likely to occur only at doses that are fetotoxic or maternally toxic (USDA, 1998[176]).

## Overall Conclusions and Perspectives

There is no doubt that pesticides influence the host's energy metabolism, nervous, cardiovascular, and endocrine systems, either directly or indirectly. It is clear that they cause many diseases, including metabolic syndrome, malnutrition, atherosclerosis, inflammation, pathogen invasion, nerve injury, and infectious disease susceptibility. Moreover, the listed pathologies may be aggravated in animals after exposure to pesticides. In this review, we discussed the classification of pesticides and their possible effects on non-target organisms. We further emphasized the importance and necessity of considering non-target living organisms as toxicological indicators for environmental pollution by pesticides. In the future, more studies should focus on the mechanisms of pesticide long-term influences on health.

Pesticides are known to exert specific effects on some cellular processes and/or key proteins involved in the regulation of general metabolism, cell growth, differentiation, and survival. Individual pesticides can influence the mitochondrial respiratory chain, leading to apoptosis and/or increased ROS levels. The latter, which is also part of the detoxifying process, may provoke inflammation and/or alter cell signaling cascades involved in the control of growth and survival or induce DNA damage. Moreover, the chronic duration of exposure to most contaminants may add another level of complexity that must also be considered in risk evaluation. Future studies should be directed to minimize or eliminate influence of pesticides on non-target living organisms, produce more specific pesticides and using of modern technologies to decrease contamination of food and other goods by pesticides.

## Acknowledgements

We thank the many members of the V. Lushchak's laboratory who have contributed over the years: Drs. T. Bagnyukova, O. Kubrak, O. Lushchak, N. Semchuk (Mosiichuk), H. Semchyshyn, O. Vasylkiv, and I. Maksymiv, the research reviewed in this article. The works were partially supported by the grants from Ministry of Education and Science of Ukraine to VIL and a discovery grants from the Natural Sciences and Engineering Research Council of Canada to KBS. 

## Conflict of interest

The authors declare that they have no conflict of interest.
